# Bati Butter as a Potential Substrate for Lipase Production by *Aspergillus terreus* NRRL-255

**DOI:** 10.3390/foods12030564

**Published:** 2023-01-27

**Authors:** Karen dos Santos Barros, Cristiane Fernandes de Assis, Millena Cristiane de Medeiros Bezerra Jácome, Wendell Medeiros de Azevedo, Adriana M. Zanbotto Ramalho, Everaldo Silvino dos Santos, Thaís Souza Passos, Francisco Canindé de Sousa Junior, Karla Suzanne Florentino da Silva Chaves Damasceno

**Affiliations:** 1 Health Sciences Center, Nutrition Postgraduate Program, Department of Nutrition, Federal University of Rio Grande do Norte, Natal 59078-900, Brazil; 2 Health Sciences Center, Nutrition Postgraduate Program, Department of Pharmacy, Federal University of Rio Grande do Norte, Natal 59012-570, Brazil; 3 Department of Chemical Engineering, Federal University of Rio Grande do Norte, Natal 59078-900, Brazil; 4 Agricultural School of Jundiaí, Laboratory of Animal Nutrition, Federal University of Rio Grande do Norte, Macaíba 59280-000, Brazil

**Keywords:** *Ouratea parviflora*, solid-state fermentation, experimental design, enzyme

## Abstract

This study evaluated bati butter (*Ouratea parviflora*) as a substrate for lipase production by solid-state fermentation (SSF) using *Aspergillus terreus* NRRL-255. A gas chromatograph with a flame ionization detector determined the bati butter fatty acid profile. Lipase production and spore count were optimized using a 3^2^ experimental design and evaluated using the response surface methodology. Moreover, the crude enzyme extract was evaluated against different pH, temperature, and activating and inhibitors reagents. Regarding the fatty acids identified, long-chain accounted for 78.60% of the total lipids. The highest lipase production was obtained at 35 °C and 120 h of fermentation, yielding 216.9 U g^−1^. Crude enzyme extract presented more significant activity at 37 °C and pH 9. β-Mercaptoethanol increased the enzyme activity (113.80%), while sodium dodecyl sulfate inactivated the enzyme. Therefore, bati butter proved to be a potential substrate capable of inducing lipase production by solid-state fermentation.

## 1. Introduction

*Ouratea* species are characterized by generally showy flowers, often yellow, with fruit development between February and March. From the seeds of *Ouratea parviflora*, bati butter can be obtained. It is aromatic with a sweet characteristic, used in preserves and seasonings, and rich in essential fatty acids [1]. Bati butter becomes interesting given the constant search for new vegetable sources of fats, attributed mainly to the fact that these materials are obtained from natural sources. Therefore, it can be used as important raw material, such as a substrate for producing enzymes (lipase) for the chemical, pharmaceutical, and food industries [2].

Lipases are enzymes belonging to the class of hydrolases that catalyze the breakdown of triacylglycerols, generating free fatty acids, diacylglycerols, monoacylglycerols, and glycerol [3]. Lipases catalyze lipids’ hydrolysis, interesterification, and esterification reactions and can be used as detergents [4]. Additionally, they are widely used in medicine [5] and the food industry [6], and as diagnostic tools in the pharmaceutical and cosmetics industry [7].

Fungi are essential lipase producers for industrial applications, as they produce the extracellular enzyme facilitating the extraction of the fermentation medium [8]. Lipase production by fungi generally uses olive oil as an inducing agent [9,10]. The enzyme produced by *A. terreus* using cacay butter, a vegetable fat, showed higher activity and potential as an inducing agent for lipase production than common inducers such as olive oil [11]. The bati butter, extracted from the seeds of *Ouratea parviflora*, is a fat composed of saturated and unsaturated fatty acids, including essential fatty acids such as oleic, palmitic, and linoleic acid [12]. Due to these characteristics, bati butter can be an alternative for lipase production since vegetable fats are a substrate for the production of lipases by microorganisms [11,13]. Moreover, the most significant interest is oils and fats containing long-chain fatty acids [14].

Lipase production can occur by submerged (FS) and semi-solid (FSS) fermentation. FS has some advantages, such as high homogeneity of the culture medium and easier control of the pH and temperature parameters [15]. The solid-state fermentation process (SSF) is an alternative for microbial enzyme production due to using agroindustrial residues and products with nutrients that induce the microorganism’s growth [16]. The plant sources are of more significant interest for their use as a substrate for lipase production, highlighting olive oil [17], palm oil [18], and soybean oil [19]. Solid-state fermentation is a process of a microorganism culture in insoluble substrates with low or without the addition of water [20,21]. Several fungi have been studied for lipase production [21]. Among the microorganisms of interest for enzyme production, *Aspergillus terreus* is reported in the literature as an excellent lipase producer [22,23,24,25].

The market value of microbial lipases was estimated at US$425.0 million in 2018 and is expected to reach US$590.2 million in 2023 [26]. For industrial applications, lipases must be produced on a large scale by fermentation by microorganisms [27]. Hence, their importance for application in the food industry tends to increase, and so scientists are looking for simple and low-cost methods [27]. Thus, this work aimed to study the potential of bati butter in the lipase enzyme production by *Aspergillus terreus* in semi-solid-state fermentation. 

## 2. Materials and Methods

### 2.1. Bati Butter

The bati (*Ouratea parviflora*) butter was donated by Plantus^®^ Ltd. (Nísia Floresta, Brazil). The samples were received in plastic containers, transported under refrigeration and light protection, and kept at 4 °C until the analyses were carried out. Before each investigation, the bati butter was melted using a water bath (QUIMIS, Q334M-28, Diadema, Brazil) at 40 °C. 

### 2.2. Methods 

#### 2.2.1. Determination of the Fatty Acids

According to Hartman and Lago [28], methyl esters were initially obtained. The samples were analyzed using a Gas Chromatography system (Thermo Scientific, CG/FID-FOCUS, Milan, Italy) with a flame ionization detector (FID) and capillary column (SUPELCO sp2560) of 100 mm × 0.25 mm × 0.2 μm. Nitrogen gas was used as a mobile phase under a flow of 2.5 mL min^−^^1^. The increase in temperature in the column was 40 °C for 3 min, 180 °C for 5 min (10 °C min^−^^1^), and 240 °C for 25 min (20 °C min^−^^1^), and the injector and detector temperatures were 230 °C and 270 °C, respectively. The injected sample volume was 1.0 μL with a split ratio of 10:1 (*v/v*), and the resulting peaks were compared to the fatty acid standards (Supelco™ FAME MIX component).

#### 2.2.2. Lipase Production

##### Microorganism

*Aspergillus terreus* (NRRL-255) was obtained from the collection of microorganisms “Agriculture Research Service Culture Collection” (Peoria, USA). It was maintained under refrigeration (4 °C) in Petri plates using a potato dextrose agar medium (PDA) and the periodical sampling technique. Before fermentation, the microorganism was activated for 168 h using a new PDA medium. An initial spore density of 1 × 10^7^ spores.mL^−^^1^ [25] was used for all experiments.

##### Fermentation

The culture medium to promote the *Aspergillus terreus* growth and lipase production by solid-state fermentation (SSF) contained: NaNO_3_ (0.3% *w/v*), MgSO_4_ (0.05% *w/v*), KCl (0.05% *w/v*), FeSO_4_ (0.001% *w/v*), KH_2_PO_4_ (0.1%, *w/v*), agar-agar (3%, *w/v*), and bati butter (25% *w/w*), pH 5.4. The medium was autoclaved (Prismatec, Itu, Brazil) for 15 min at 121 °C and placed in a Petri plate (90 × 15 mm) [29], followed by inoculation with a suspension of 10^7^ spores.mL^−^^1^.

##### Design of the Experiment

Lipase production and spore count were optimized using a full 3^2^ experimental design with repetitions, totaling 18 assays. The independent variables were temperature (°C) and fermentation time (h). The dependent variables were spore count (log) and enzyme activity (U g^−^^1^). Each independent variable was evaluated at three levels (temperature: +1 = 45 °C, 0 = 35 °C, −1 = 25 °C and time: +1 = 168 h, 0 = 120 h, −1 = 72 h).

#### 2.2.3. Enzyme Extraction

The crude enzyme extract was obtained according to Jain and Naik [11] with modifications. Fifty milliliters of Tris-HCl (0.05 M, pH 8.0) was added to an Erlenmeyer flask and homogenized using a shaker (QUIMIS, Q816M20, Hangzhou Zhejiang, China) at 37 °C for 30 min. The contents of the Erlenmeyer were filtered and then centrifuged (CENTRIBIO, 80-2B, São Paulo, Brazil) at 5240× *g* for 10 min. The supernatant was removed, and the activity (%) of the crude enzyme extract was analyzed immediately.

#### 2.2.4. Determination of Enzyme Activity

The enzyme activity was determined by spectrophotometry using ρ-nitrophenyl palmitate (*ρ*NPP) as a substrate. Ten microliters (10 µL) of the enzyme extract and 90 μL of the substrate were added to a microplate, following the incubation at 37 °C for 30 min. The enzyme activity was determined using a microplate reader (Biochrom Asys, UVM340, Cambridge, UK) at 410 nm [20]. Ten microliters of Tris-HCl (0.05 M, pH 8) and 90 μL of the substrate were used as a blank.

A unit (U) of enzyme activity was defined as the number of enzymes releasing 1 μmol of *ρ*NPP per minute. Lipase activity was expressed in U g^−^^1^ of the substrate used for SSF.

#### 2.2.5. Spore Count

Spore count estimated the fungal growth in a Neubauer chamber. It was expressed in spores.mL^−^^1^ and transformed to log.

#### 2.2.6. Lipase Characterization

##### Effects of Temperature and pH

The temperature and pH effects were evaluated using the crude enzyme extract. The temperature ranged from 35 °C to 75 °C, while the pH range was from 4.0 to 11.0. The buffers used were phosphate-citrate (pH 4.0 to 7.0), Tris-HCl (pH 8.0), and glycine-NaOH (pH 9.0 to 11.0). The relative activity was calculated using the temperature of 37 °C and pH 8.0 as a control.

##### Effects of Inhibitory and Activating Agents

The agents used to evaluate the inhibitory or activating activity of the crude enzyme extract were NH_4_Cl, (NH_4_)_2_SO_4_, CaCl_2_, CuSO_4_, FeCl_2_, FeSO_4_, MgSO_4_, K_2_SO_4_, KI, ZnSO_4_, Na_2_SO_4_, NaCl (metal ions), glycerol, β-mercaptoethanol (reducing agent), sodium dodecyl sulfate (SDS)–(anionic surfactant), EDTA (chelating agent), Triton X-100 (non-ionic surfactant). A concentration of 1.0 mM was used for all agents except for urea (2.0 mM) (Dinâmica, Indaiatuba, Brazil). The relative activity was calculated using the Tris-HCl buffer (0.05 M) as a control.

#### 2.2.7. Statistical Analysis

The experimental runs were performed randomly. The data were analyzed, and the statistical significance of the second-order polynomial model was determined by the F-test (ANOVA). The response surface was plotted using Statistica 7.0 (StatSoft Inc., Tulsa, OK, USA).

The results obtained to determine the crude extract’s enzyme activity in different conditions (pH and temperature) were submitted to ANOVA, followed by Dunnet’s post hoc test to evaluate the relative activities concerning the control. A *p*-value < 0.05 was adopted to determine significant differences, and the statistical analyses were performed in XLSTAT software version 2018.5 (Addinsoft, Paris, France).

## 3. Results and Discussion 

### 3.1. Determination of Fatty Acids of Bati Butter

The chemical determination allowed the identification of fourteen fatty acids in bati butter (Table 1).

It was observed that the saturated (51.54%) and unsaturated (48.20%) fatty acid percentages present in the butter were close, and the predominant fatty acids were palmitic (33.50%), oleic (28.64%), linoleic (14.56%), and myristic (14.51%). These data corroborate the results demonstrated by Galvão et al. [12], who investigated *Ouratea* sp. butter. It is important to emphasize the presence of long-chain fatty acids (C14–C24), which favors bati butter as a substrate for lipase production by fungi, to improve enzyme production [14,30]. Frota et al. (2021) [31] analyzed the fatty acids of batiputá oil extracted from *Ouretea fieldingiana*, finding predominantly palmitic (24.55%), oleic (30.81%), and linoleic (20.02%) acids.

### 3.2. Lipase Production

Lipase production took place in Petri dishes, where the bati butter was placed in a medium enriched with salts using agar to promote the solidification of the culture medium. In this way, it was possible to verify if the bati butter provides a favorable environment for the growth of the microorganism and enzyme production. It should be noted that there are no reports in the literature on the production of lipase by *Aspergillus terreus* and bati butter. Vegetable oils can induce the growth of *Aspergillus* sp., promoting a high concentration of lipase [25]. Azevedo et al. [11] analyzed different substrates to produce the lipase enzyme by the fungus *A. terreus*. They found that cacay butter induced lipase production similar to olive oil.

The effects of temperature and fermentation time on spore count and enzyme activity were evaluated using the experimental design [32]. According to the adopted experimental condition, the spore count varied between 7.20 ± 0.17 and 8.1 ± 0.08 log, and the enzyme activity was between 0 and 216.9 U g^−^^1^ (Table 2).

The highest activity was found at the central levels of the tested factors (35 °C/120 h of fermentation). Combined with the evaluation of the fatty acids of bati butter, these results suggest a high potential of this as a substrate for lipase production by *A. terreus* using the solid-state fermentation process. It also shows a high lipase production compared to the study by [22], which produced lipase by *A. terreus* using maize oil as a substrate and lipase production in the range from 25 °C to 45 °C with a maximum yield of 70.1 U g^−^^1^ at 37 °C in 96 h.

The estimated effect for each variable and their interactions was determined and reported in Table 3. In the 95% confidence interval, temperature (Quadratic term-Q) and fermentation time (Linear term-L) had a positive and significant effect on enzyme activity (Table 3). Regarding the spore count, only the time variable (Quadratic term) did not significantly influence it.

A second-order polynomial model was generated to describe the enzyme activity (Log EA) (Equation (1)) and the spore count (Log S) (Equation (2)) as a function of independent variables (factors), temperature (X_1_), and fermentation time (X_2_).

(1) $$\mathrm{Log}\;[\mathrm{EA}(U\;g^{-1})+1]=1.937842+0.186031\;X_{1}+1.06041\;X_{2}\;–\;0.420417\;X_{1^{2}}\;–\;0681606\;X_{2^{2}}$$

(2) $$\mathrm{Log}\;S=7.86444\;–\;0.15\;X_{1}+0.24\;X_{2}\;–\;0.16667\;X_{1^{2}}\;–\;0.09667\;X_{2^{2}}\;–\;0.20\;X_{1}\;X_{2}\;–\;0.1657\;X_{1^{2}}\;X_{2}\;$$

According to the analysis of variance (Table 4), the F values calculated for the models of enzyme activity (Equation (1)) and spore count (Equation (2)) were 7.53 and 24.00, respectively. The results were higher than the tabulated F_4.13_ value (3.18) in the 95% confidence interval. Thus, the models can be considered statistically significant, according to the F-test. Whereas the lack of fit was significant for enzyme activity, it showed a very low pure error. 

**Figure 1:**
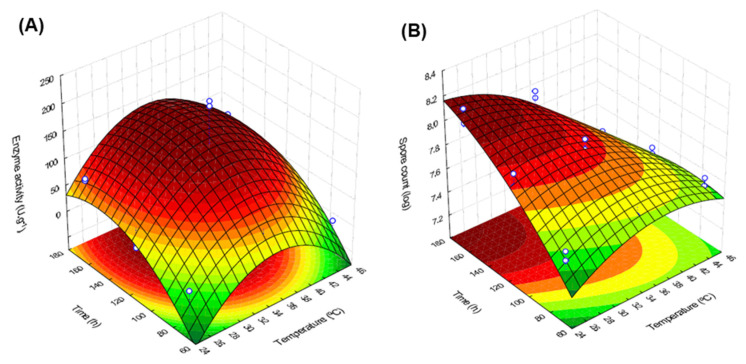
Response surface of the variables temperature and fermentation time on the response of enzyme activity (**A**) and spore count (**B**).

For the enzyme activity, the response surface (Figure 1) showed a well-defined region for maximum enzyme activity. The optimal conditions were found by determining the critical point (derivative equation to zero) of Equation (1). The maximum enzyme activity was reached at 37.2 °C and during the cultivation time of 157 h (Figure 1A). On the other hand, a higher number of spores were obtained in extreme conditions (Figure 1B), with lower temperature (29.7 °C) and longer fermentation time (168 h).

It can be seen that the maximum enzyme activity under the conditions studied was higher at a temperature above, which is reported in some studies as the ideal temperature range (28–32 °C) for the production of lipase by fungi [8,25]. However, it is important to consider that it will have an ideal temperature for each microorganism, which will be defined according to other factors such as substrate, fermentation time, and pH [33,34].

Fleuri et al. [35] tested several strains of fungi related to lipase production. They observed that *Aspergillus* sp. produced lipase with higher enzyme activity using wheat bran, soy bran, and wheat bran combined with sugarcane bagasse as substrates for 96 h.

Sethi et al. [25] used the mustard cake to produce lipase at 30 °C for 96 h by the fungus *Aspergillus terreus* and obtained an enzyme activity of 525 U g^−1^ with substrate supplemented with palm oil. However, several studies report an optimal activity of the enzyme produced by *A. terreus* at a temperature above 32 °C, as found in the present study.

Gulati et al. [23] evaluated the lipase production by *A. terreus* in a medium containing maize oil and casein, obtaining the maximum yield at 37 °C in 96 h of culture. Mahmoud et al. [24] obtained an enzyme activity of 15.46 U.mL^−1^ produced by *A. terreus*, during a fermentation period of 144 h at 45 °C, with hydrocarbons as a substrate.

Thus, it is observed that the substrate used in the present study has the potential for lipase production due to the good enzyme activity obtained. This may be related to the long-chain fatty acid composition of bati butter, which favored the microorganism growth. Positive results encourage further studies using bati butter to promote lipase production.

### 3.3. Lipase Characterization

#### 3.3.1. Temperature and pH Effect on Lipolytic Activity

Temperature is an important variable in enzyme-catalyzed esterification due to its effect on enzyme activity, substrate solubility, solution viscosity, and reaction medium [36]. 

The enzyme was incubated at 35 °C to 75 °C at pH 8.0 to evaluate the temperature effect on lipase activity. The temperature of 37 °C was considered the control because the more significant activity at this condition was observed. Kamini et al. [37] used *A. terreus* to produce lipase, finding the optimal temperature of 35 °C for enzyme activity, similar to the results found in the present study.

**Figure 2:**
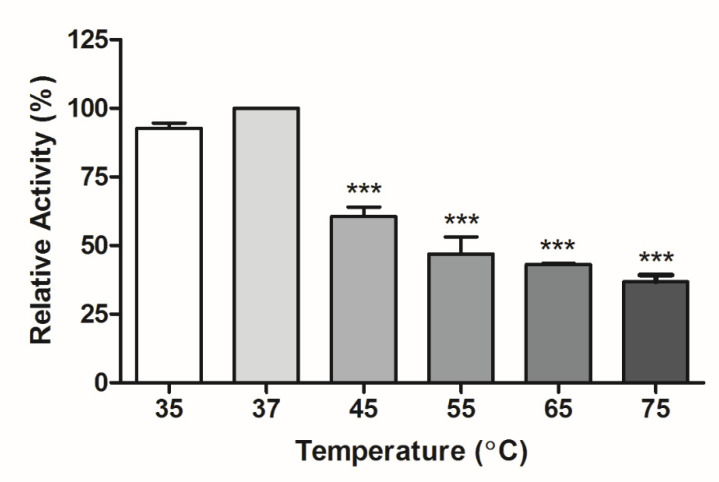
Effect of temperature on the enzyme activity of lipase. *** Significantly different values of control (37 °C), according to ANOVA followed by Dunnett’s post hoc test (*p* < 0.0001). Mean and standard deviation.

The results (Figure 2) showed that the different temperatures evaluated, compared to the control (37 °C), significantly influenced (*p* < 0.0001) the relative enzyme activity. There was a decrease in the relative activity as a function of the temperature increase, significantly affecting the range from 45 °C to 75 °C (*p* < 0.0001).

At high temperatures or other extreme conditions, non-covalent forces decrease (except for the hydrophobic interactions), and the enzyme loses activity due to the disordered conformation [38]. In the native three-dimensional protein structure, the active center has several amino acids close to each other. Thus, the unfolding results in the activity loss of the enzyme’s active site. Azevedo et al. [11] reported similar results. They found the maximum enzyme activity for lipase produced by *A. terreus* in cacay butter and wheat bran at 35 °C, also noting that the increase in temperature decreased the activity due to enzymatic denaturation. 

Hamdy; Abo-Tahon [39] used *A. terreus* for lipase production from a medium supplemented with olive oil and identified maximum enzyme activity at 30 °C after 96 h. Utami et al. [10] found that the enzyme activity of lipase produced by *A. niger*, using rice bran supplemented with olive oil as a substrate, showed satisfactory results at 30 °C. Additionally, they observed a considerable relative activity reduction as a function of the temperature increase evaluated, according to the range from 30 to 90 °C. Sethi et al. [25] also assessed the influence of temperature on the enzyme activity produced by *A. terreus* and verified that the highest enzyme activity at 50 °C and higher temperatures led to enzymatic denaturation, promoting the decrease in activity.

**Figure 3:**
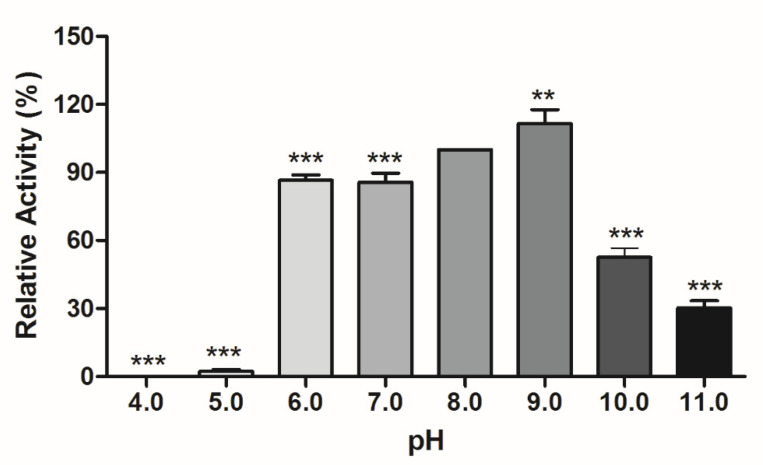
Effect of pH on lipase activity. ** Values significantly different from control (pH 8), according to ANOVA followed by Dunnett’s post hoc test (*p* < 0.001). *** Values significantly different from control (pH 8), according to ANOVA followed by Dunnett’s post hoc test (*p* < 0.0001). Mean and standard deviation.

Several pH values were tested (Figure 3) to evaluate the influence of pH on the lipase activity produced by *A. terreus* using bati butter as a substrate. It is important to note that pH 8 was used as a control since it was used on the enzyme activity assays performed.

The enzyme produced by *Aspergillus terreus* presented good relative activity at pH 6 to 9. Compared to the control, all the pH tested significantly influenced (*p* < 0.001; *p* < 0.0001) the relative enzyme activity. At pH 9, it can be observed that an increase in the enzyme activity exceeded 100% of the control activity. Similar behavior was found by Azevedo et al. [11] for the lipase produced by the same strain of *A. terreus* used in the present study. 

Sethi et al. [25] carried out the enzymatic characterization that showed that the lipase obtained from *A. terreus* was significantly active in the pH range from 6 to 9, as in the present study, but with an optimum pH 6.0, becoming unstable in pH values above 9.0 and below 6.0.

The pH plays an essential role in hydrolysis reactions catalyzed by enzymes. The pH variation can be related to the possible conformational changes, ionization state alterations, and protein dissociation in the reactive medium (macroenvironment). The stability characteristics are of considerable interest in industrial applications because of production [40]. The pH range of the crude enzyme extract presented shows its potential for application in the food, pharmaceutical, or chemical industry.

#### 3.3.2. Effect of Inhibitors and Activating Agents on Lipolytic Activity

The enzyme activity was evaluated in the presence of inhibitors and activating agents. Table 5 shows the agents that significantly affected (*p* < 0.05) the enzyme activity. It is possible to note that β-mercaptoethanol increased the activity of the protein (*p* < 0.05). 

Pastore et al. [41] identified a similar increase in the relative activity of lipase produced by *Rhizopus* sp. β-mercaptoethanol is a chemical compound commonly used to reduce disulfide bonds (S-S). Therefore, it can be suggested that this compound acted as an antioxidant neutralizing the oxidative effect of the S-S bond formed between the cysteine residues. It could act as a biological antioxidant in enzymatic reactions, obtaining a higher enzyme activity [36]. On the other hand, Sethi et al. [25] observed that the action of lipase produced by *A. terreus* was reduced by more than 77% when treated with β-mercaptoethanol. The other compounds decreased the enzyme activity, emphasizing SDS by reducing enzyme activity to zero. However, the agents NH_4_Cl, (NH_4_)_2_SO_4_, Na_2_SO_4_, and NaCl, despite reducing the enzyme activity, did not present a statistically significant difference (*p* > 0.05) compared to the control.

Gururaj et al. [42] produced lipase from *Acinetobacter* sp. and observed that the Zn, Mg, Ca, Fe, and Mn ions decreased enzyme activity, showing that it does not require metallic ions to promote the activity. Furthermore, the enzyme’s inhibitory nature may be related to the metals’ interaction with the lipase structure’s side chain groups, influencing the conformation and stability [43].

According to Gururaj et al. [42], there was an activity decrease in EDTA, Triton X-100, and SDS, corroborating with the present study’s findings. EDTA is an organic compound that acts as a chelating agent, forming stable complexes with several metallic ions. The decrease in activity related to the presence of EDTA can be explained by the interaction of ions, which can be bound to the enzyme’s active site, promoting activity reduction [44]. However, Sethi et al. [25] observed that EDTA did not alter the activity and stability of lipase from *A. terreus* using mustard oil as a substrate, showing that this result may vary according to the substrate evaluated.

Triton X-100 (52.06 U g^−1^) and SDS (0.00 U g^−1^) are non-ionic detergents that interact with lipase through hydrophobic bonds [32]. These interactions may promote changes in the lipase structural conformations, decreasing enzyme activity [45].

## 4. Conclusions

This is the first study showing lipase production by the fungus *Aspergillus terreus* using bati butter as a substrate. Bati butter could be used as a substrate to promote extracellular lipase production from *A. terreus*. Moreover, it showed stability against some metal ions. Thus, the crude enzyme extract from bati butter can be considered a biotechnological product with potential added value.

## Figures and Tables

**Table 1:** The fatty acid composition of bati butter.

Fatty Acids	%
Capric acid (C10:0)	0.84
Lauric acid (C12:0)	2.00
Tridecanoic acid (C13:0)	0.07
Myristic acid (C14:0)	14.51
Pentadecanoic acid (C15:0)	0.16
Palmitic acid (C16:0)	33.50
Heptadecanoic acid (C17:0)	0.27
Stearic acid (C18:0)	0.19
Elaidic acid (C18:1)	4.37
Oleic acid (C18:1, *w*-9)	28.64
Linoleic acid (C18:2, *w*-6)	14.56
Linolelaidic acid (C18:2, 6t)	0.10
Eicosanoic acid (C20:1)	0.42
Eicosadienoic acid (C20:2)	0.11
SFA	51.54
UFA	48.20
MUFA	33.43
PUFA	14.77

SFA: saturated fatty acids; UFA: unsaturated fatty acids; MUFA: monounsaturated fatty acids; PUFA: polyunsaturated fatty acids.

**Table 2:** Experimental design to evaluate the effects of the independent variables temperature (°C) and fermentation time (h) on spore count (log) and enzyme activity (U g^−1^) of the lipase produced from *Aspergillus terreus* NRRL-255 by solid-state fermentation (SSF).

		Coded Levels (Real Values)			
Assay	Repetition	X_1_	X_2_	Spore Count * (log)	Enzyme Activity * (U g^−1^)
1	1	−1 (25)	−1 (72)	7.50 ± 0.06	0.00 ± 0.00
2	1	0 (35)	1 (168)	7.98 ± 0.04	129.08 ± 1.63
3	1	1 (45)	0 (120)	7.60 ± 0.04	28.97 ± 0.81
4	1	0 (35)	−1 (72)	7.51 ± 0.03	0.00 ± 0.00
5	1	1 (45)	−1 (72)	7.60 ± 0.05	0.00 ± 0.00
6	1	−1 (25)	1 (168)	8.10 ± 0.08	68.74 ± 0.81
7	1	−1 (25)	0 (120)	7.80 ± 0.07	16.90 ± 1.63
8	1	1 (45)	1 (168)	7.20 ± 0.17	72.76 ± 3.25
9	1	0 (35)	0 (120)	7.82 ± 0.03	216.90 ± 0.001
10	2	0 (35)	1 (168)	8.04 ± 0.04	133.10 ± 0.81
11	2	1 (45)	0 (120)	7.50 ± 0.04	33.56 ± 5.69
12	2	−1 (25)	−1 (72)	7.50 ± 0.06	0.00 ± 0.00
13	2	1 (45)	−1 (72)	7.50 ± 0.05	0.00 ± 0.00
14	2	0 (35)	−1 (72)	7.55 ± 0.03	0.00 ± 0.00
15	2	−1 (25)	1 (168)	8.00 ± 0.08	72.19 ± 0.81
16	2	−1 (25)	0 (120)	7.90 ± 0.07	9.43 ± 0.81
17	2	1 (45)	1 (168)	7.40 ± 0.17	77.36 ± 3.25
18	2	0 (35)	0 (120)	7.90 ± 0.03	208.28 ± 2.44

* Mean ± Std Dev (*n* = 2). X_1_: Temperature (°C); X_2_: Time (h).

**Table 3:** Estimated effects of the variables temperature (X_1_) and time (X_2_) on enzyme activity and spore count.

Independent Variable and Interaction	Enzyme Activity (EA)		Spore Count (LogS)	
	Estimated Effect	*p*-Value	Estimated Effect	*p*-Value
Mean/intercept	59.29 *	0.000000 *	7.69 *	0.000000 *
X_1_ (L)	7.57 *	0.003473 *	−0.32 *	0.000053 *
X_1_ (Q)	82.90 *	0.000000 *	0.16 *	0.002530 *
X_2_ (L)	92.20 *	0.000000 *	0.28 *	0.000177 *
X_2_ (Q)	39.57 *	0.000000 *	0.08	0.071984
X_1_ (L) × X_2_(L)	2.30	0.355445	−0.40 *	0.000044 *
X_1_ (Q) × X_2_(L)	29.17 *	0.000000 *	0.15 *	0.010254

* Statistically significant values (*p* < 0.05).

**Table 4:** Analysis of variance (ANOVA) to validate the models (Equations (1) and (2)).

Source of Variation	Square Sum	Degree of Freedom	Mean Square	F-Value
Enzyme activity Equation (1)				
Regression	59,429.97	4	14,857.493	7.53
Residual	25,645.99	13	1972.769	
Lack of fit (*p* < 0.05)	25,545.76	4		
Pure error	100.24	9		
R^2^	0.70			
Spore Count Equation (2)				
Regression	0.97	4	0.242	22.00
Residual	0.14	13	0.011	
Lack of fit	0.09	4		
Pure error (*p* = 0.06)	0.05	9		
R^2^	0.89			
Listed F-value (95%)				F_4.13_ = 3.18

**Table 5:** Effect of activator and inhibitor agents on lipase activity produced from bati butter.

Activators/Inhibitors	Relative Activity (%) Mean (SD)
NH_4_Cl	94.17 ± 0.81
(NH_4_)_2_SO_4_	95.00 ± 7.09
CaCl_2_	75.02 * ± 6.10
CuSO_4_	48.18 * ± 2.29
FeCl_2_	16.75 * ± 2.19
FeSO_4_	32.53 * ± 1.74
MgSO_4_	48.11 * ± 1.70
K_2_SO_4_	82.28 * ± 3.26
KI	91.56 * ± 1.52
ZnSO_4_	48.67 * ± 0.93
Na_2_SO_4_	94.77 ± 2.76
NaCl	96.43 ± 2.14
Triton X-100	52.06 * ± 1.79
*β*-mercaptoethanol	113.80 * ± 3.24
Urea	80.26 * ± 8.12
EDTA	40.53 * ± 0.70
SDS	0.00 *
Glycerol	50.42 * ± 2.23
Control	100

* Mean ± Std Dev (*n* = 4). * Relative enzyme activity was evaluated according to Dunnett’s post hoc test (*p* < 0.05).

## Data Availability

The datasets generated for this study are available on request to the corresponding author.
